# Sparse Reconstruction of Pressure Field for Wedge Passive Fluidic Thrust Vectoring Nozzle

**DOI:** 10.3390/s26030811

**Published:** 2026-01-26

**Authors:** Zi Huang, Yunsong Gu, Qiuhui Xu, Linkai Li

**Affiliations:** 1 College of Aerospace Engineering, Nanjing University of Aeronautics and Astronautics, Nanjing 210016, Chinalinkaili@nuaa.edu.cn (L.L.); 2 Tianyin Lake Science and Technology Innovation College, Nanjing Institute of Technology, Nanjing 211167, China

**Keywords:** fluidic thrust vectoring, pressure-field reconstruction, compressed sensing, POD modal decomposition

## Abstract

Fluidic thrust vectoring control (FTVC) enables highly agile flight without the mechanical complexity of traditional vectoring nozzles. However, a robust onboard identification of the jet deflection state remains challenging when only limited measurements are available. This study proposes a sparse reconstruction of the pressure field method for a wedge passive FTVC nozzle and validates the approach experimentally on a low-speed jet platform. By combining the proper orthogonal decomposition (POD) algorithm with an $l_{1}$-regularized compressed sensing method, a full Coanda wall pressure distribution is reconstructed from the sparse measurements. A genetic algorithm is then employed to optimize the wall pressure tap locations, identifying an optimal layout. With only four pressure taps, the local pressure coefficient errors were maintained within |ΔCp| < 0.02. In contrast, conventional Kriging interpolation requires increasing the sensor count to 13 to approach the reconstruction level of the proposed POD–compressed sensing method using 4 sensors, yet still exhibits a reduced fidelity in capturing key flow structure characteristics. Overall, the proposed approach provides an efficient and physically interpretable strategy for pressure field estimation, supporting lightweight, low-maintenance, and precise fluidic thrust vectoring control.

## 1. Introduction

Thrust vectoring control (TVC) is a key technology for new-generation high-performance fighter aircraft, offering significant improvements in maneuverability and air combat effectiveness. According to the mechanism of jet deflection, TVC systems can generally be classified into mechanical and fluidic types. Mechanical TVC achieves jet deflection by altering the nozzle’s structural geometry. However, such designs usually feature complex structures and multiple moving components, resulting in an increased system weight and potential degradation of the aircraft’s stealth characteristics. In contrast, fluidic thrust vectoring nozzles achieve jet deflection through flow-control mechanisms without changing the outer geometric shape of the nozzle [1], offering advantages in structural simplicity, reduced mass, and improved stealth performance. Studies of NASA’s Fluidic Injection Nozzle Technology (FLINT) have demonstrated that fluidic TVC (FTVC) nozzles can increase the engine thrust-to-weight ratio by approximately 7–12% and reduce maintenance costs by 37–53% compared with traditional mechanical nozzles, indicating a strong engineering applicability [2,3,4].

For mechanical TVC nozzles, the jet deflection state is closely related to the deflection angle of the mechanical components [5]. However, the jet deflection of FTVC nozzles is mainly governed by flow-control actuation parameters [6]. Some FTVC configurations even exhibit thrust vector angle hysteresis or abrupt jumps, where the same control input may correspond to multiple possible jet deflection states [7], making it impropriate to infer the jet deflection directly from the control commands, particularly for the passive FTVC nozzles, which control jet deflection by changing the opening rate between the Coanda surface secondary flow channels and the ambient air through secondary flow valves. The resulting jet deflection process exhibits a two-stage transmission mechanism: valve displacement first modifies the pressure distribution within the secondary flow channels, after which the upper–lower secondary flow channel pressure difference drives the main jet to deflect. This process contains two nonlinear mapping stages, from valve displacement to pressure change and from pressure difference to jet deflection, making it difficult to estimate the actual jet deflection state simply from the valve displacement. Consequently, the accurate perception and control of jet deflection remain challenging.

At present, the measurement of the deflection state for FTVC nozzles primarily relies on ground experiments or wind tunnel testing. Common measurement techniques include force balance measurements and flow-field vector measurement methods such as particle image velocimetry (PIV) [8], schlieren imaging [9] and wall pressure integration [10]. However, force balances are impractical for onboard use, while optical methods require a dedicated optical path and strict operating conditions, making them unsuitable for airborne measurements. In contrast, pressure sensors exert minimal structural influence, are easy to integrate and maintain, and therefore offer a strong potential for engineering applications. The deflection force arises primarily from momentum conservation. For passive FTVC nozzles [11,12], since the momentum contribution of the secondary flow is negligible, the thrust vector angle mainly originates from the pressure difference acting on the Coanda surfaces [13]. This means that the jet deflection state can be estimated directly through wall pressure integration [14].

To obtain the wall pressure distribution, the most direct approach is to deploy a high-density pressure sensor array along the nozzle walls. However, using an excessively large number of sensors is impractical for real applications. To address this limitation, researchers have introduced compressed sensing theory [15], which enables the accurate reconstruction of high-dimensional signals from limited sampling. Compressed sensing flow-field reconstruction methods have been validated in various flow scenarios, including airfoil flows [16,17,18] and the prediction of aircraft wall pressure [19]. When combined with temporal information, these methods can reconstruct and predict the evolution of unsteady flow fields [20,21]. Based on the reconstructed flow field, aerodynamic forces and moments can be further estimated through pressure integration, providing a foundation for aerodynamic load estimation and control. To optimize sensor placement for pressure reconstruction, the existing studies have employed various optimization algorithms, such as sensitivity analysis [22], trade-off analysis [23], and the particle swam algorithm [24]. Regarding reconstruction algorithms, current approaches include machine learning-based models [25], mathematical interpolation methods such as Kriging [26,27] and inverse distance weighting (IDW) [28], and modal decomposition techniques. Machine learning establishes nonlinear mappings between pressure and flow fields, often yielding a high accuracy but lacking physical interpretability. Interpolation methods are simple and convenient to implement, but essentially remain purely mathematical constructions that cannot capture dominant flow structures. In contrast, the modal decomposition approach used in this study extracts dominant energetic modes and represents the flow field as a linear combination of a finite set of physically interpretable modes, offering a clear physical meaning, effective dimensionality reduction, and high computational efficiency [29,30].

Although previous studies have made significant progress in theoretical development and airfoil/aircraft surface pressure distribution reconstruction validation, systematic investigations on pressure reconstruction for passive FTVC nozzles remain limited. Shi et al. [13] combined Kriging models with multi-objective optimization algorithms to design pressure tap layouts for thrust vector angle sensing in 2D passive FTVC nozzles, demonstrating the feasibility of estimating vector angles from wall pressure distributions. However, 2D nozzles inherently exhibit thrust vector angle jumps, posing challenges for practical applications [7]. The wedge passive FTVC nozzle examined in this work achieves continuous control of the thrust vector angle by introducing three-dimensional near-wall flow structures. Nevertheless, the resulting wall pressure distribution exhibits strong three-dimensional characteristics, requiring a large number of measurement points for accurate reconstructions when using conventional interpolation techniques.

To overcome these challenges, this study proposes a pressure distribution estimation method tailored to a wedge passive fluidic TVC nozzle. The method employs compressed sensing and proper orthogonal decomposition (POD) as the core framework to establish a mapping between sparse pressure measurements and the full wall pressure field, enabling the high-fidelity reconstruction of complex pressure distributions. A genetic algorithm is further used to perform an optimization of the layout of the sparse pressure sensor array, ensuring a high reconstruction and vector angle estimation accuracy, while minimizing the number of required sensors. Finally, the reconstruction results of the POD algorithm and the Kriging algorithm were compared in terms of error and flow-field structure.

## 2. Methodology

### 2.1. Experimental Setup

#### 2.1.1. Setting of Low-Speed Jet Simulation Platform

**Figure 1:**
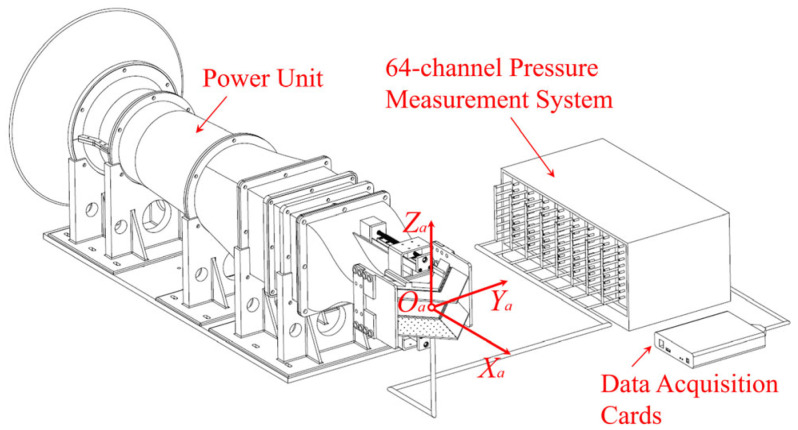
Low-speed jet simulation platform.

The experiments are conducted on a low-speed jet simulation platform in the Aerodynamics Laboratory of Nanjing University of Aeronautics and Astronautics. The power unit is a TP Power 144 mm electric ducted fan, which is powered by 48 V DC. The low-speed jet simulation platform is equipped with a pressure measurement system. As shown in Figure 1, the coordinate system $O_{a}X_{a}Y_{a}Z_{a}$ is defined as the airflow axis system. The origin $O_{a}$ is set at the center of the nozzle inlet plane; the $O_{a}X_{a}$ direction is along the airflow axis backward, and the $O_{a}Z_{a}$ direction is vertically upward. The jet velocity at the nozzle inlet $\left(V_{\infty }\right)$ is 25 m/s. With the chord length of the Coanda wall ($L_{c}$) as the reference length, the jet Reynolds number is *Re* = $1.02\times 10^{4}$. The experiment was carried out at a room temperature of 15 °C, atmospheric pressure of 102,400 Pa, and density of 1.24 kg/m^3^.

#### 2.1.2. Pressure Measurement System

Coanda wall static pressure distributions are obtained using a 64-channel measurement device developed by the Flight Measurement and Control Innovation Laboratory at Nanjing University of Aeronautics and Astronautics (NUAA). The pressure measurement system employs high-sensitivity differential pressure transducers (range: ±1034.21 Pa; accuracy: 0.05% F.S.) referenced to ambient atmospheric conditions. The measurement variance of the sensor is approximately 0.2 Pa. Analog signals are digitized via a 16-bit acquisition card operating at a sampling frequency of 1 kHz. A total of 2001 snapshots were collected per experiment. Here, δ is the differential opening of secondary flow valves.

Ten spanwise columns of pressure taps on the nozzle’s Coanda wall are used to monitor surface pressure. Each column features six equally spaced sensors. The corresponding dimensionless coordinates ($X_{b}/L_{c}$, $Y_{b}/Y_{s}$) for this topology are summarized in Table 1. Here, $X_{b}$ is the *X* coordinate of the pressure tap, $L_{c}$ is the Coanda wall chord length, $Y_{b}$ is the Y coordinate of the pressure tap, and $Y_{s}$ is twice the length of the nozzle trailing edge.

$P_{i}$ is the pressure measured by the pressure sensor, which is calculated as follows:

(1) $$P_{i}=P_{s}-P_{a}$$

where $P_{s}$ is the surface static pressure, and $P_{a}$ is the atmospheric pressure. The resulting data are reduced to dimensionless pressure coefficients $C_{p}$, according to Equation (2):

(2) $$C_{pi}=\frac{P_{i}}{0.5\cdot \rho \cdot {V_{\infty }}^{2}}$$

#### 2.1.3. Passive Fluidic Thrust Vectoring Nozzle Model

**Figure 2:**
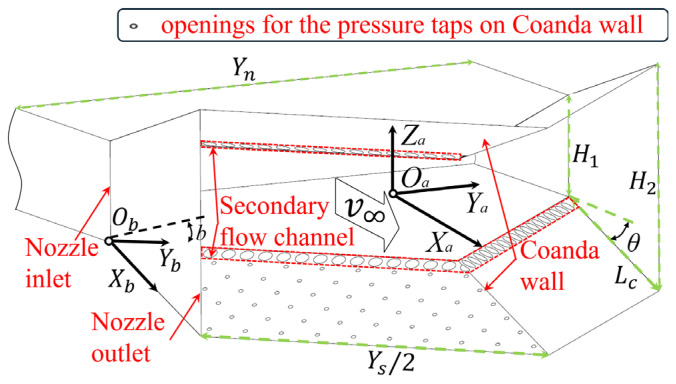
Definition of geometric parameters for wedge passive FTVC nozzle.

The schematic of the passive fluidic thrust vectoring nozzle, mounted at the outlet of a low-speed jet simulation platform, is presented in Figure 2. The nozzle assembly comprises the main nozzle structure, secondary flow channels, and Coanda walls. Regarding the geometry definitions, the Coanda wall is inclined at a deflection angle $\theta =15^{\circ }$ relative to the freestream velocity axis ($v_{\infty }$). To characterize the Coanda wall, a body-fitted coordinate system $O_{b}X_{b}Y_{b}$ is established on the Coanda wall. The origin $O_{b}$ is located at the intersection of the nozzle outlet and the Coanda wall side leading edge. The $O_{b}X_{b}$ axis aligns with the chordwise direction (streamwise), while the $O_{b}Y_{b}$ axis aligns with the leading edge. The nozzle features a trailing-edge bevel angle, denoted as b, defined as the angle between the $O_{b}Y_{b}$ axis and the $O_{a}Y_{a}$ axis. The $Y_{S}$ is twice the length of the nozzle trailing edge. The key geometric parameters of the nozzle are defined as Table 2.

**Figure 3:**
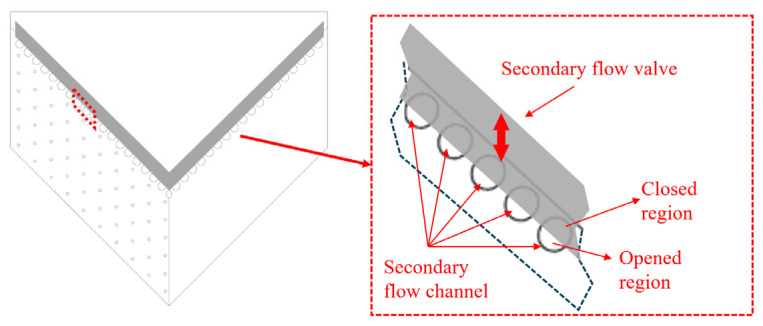
Schematic of secondary flow channel area control method (top view). The red dash square indicates the zoomed-in area.

The Coanda wall is perforated at the leading edge by a row of 4 mm diameter ports spaced 5.6 mm center-to-center. These ports serve as secondary flow channels connecting the wall surface to the ambient environment. As shown in Figure 3, flow rate through these channels is regulated via a sliding valve that translates along the $O_{a}X_{a}$ direction, ensuring synchronous adjustment across the full span. Control authority is parametrized by the differential opening of the secondary flow valve, δ. The valve kinematics, governed by Equations (3)–(5), allow for states ranging from neutral (δ=0), where both valves are obstructed to 50% capacity (δ=0), to fully vectored (δ=−1/1).

(3) $$\delta =O_{u}-O_{d}$$

(4) $$O_{u}=\frac{\delta }{2}+0.5$$

(5) $$O_{d}=-\frac{\delta }{2}+0.5$$

**Figure 4:**
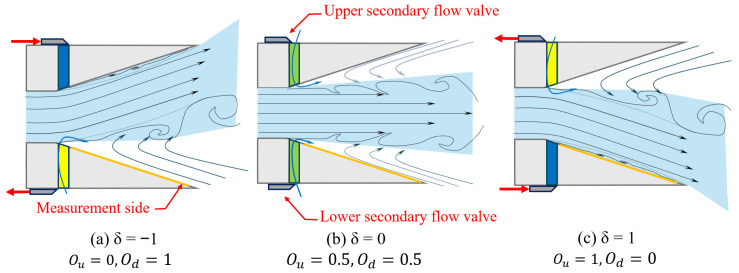
Schematic diagram of jet deflection control method for wedge passive FTVC nozzle.

The nozzle achieves vectoring authority by asymmetrically actuating control valves that link the secondary flow channels to the ambient environment. As depicted in Figure 4, the jet deflection state is governed by the openings of secondary flow valves: a synchronous opening maintains a neutral jet, which is shown in Figure 4b, while an asymmetric closure forces the jet to attach to the Coanda wall on the closed side (down side), generating jet deflection, which is shown in Figure 4c. The orange thick line indicates the pressure measurement side of the Coanda wall. When δ increases from −1 to 1, the pressure distribution of the detached state and attached states can be obtained.

### 2.2. Pressure Reconstruction Method

**Figure 5:**
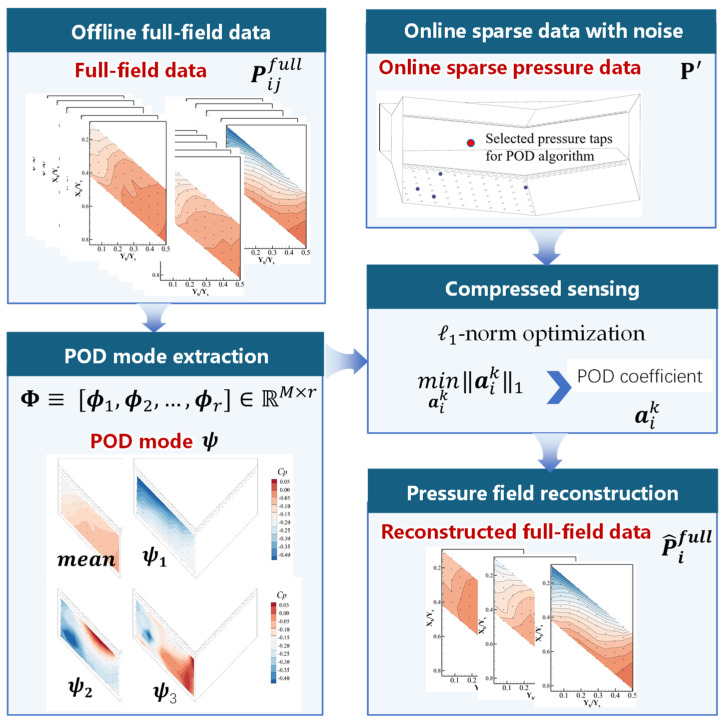
Schematic diagram of POD modal decomposition and reconstruction.

The proper orthogonal decomposition (POD) is a linear dimensionality reduction and modal extraction technique based on the intrinsic characteristics of the pressure field. Its core idea is to extract a series of energy-dominant modes from high-dimensional pressure data, and it can reconstruct the full-field pressure distribution via a linear combination of these modes. The decomposition and reconstruction process of POD is shown in Figure 5:

#### 2.2.1. Extraction of Dominant POD Modes

The original dataset is obtained from wall pressure measurements of the experiments. The control parameter δ is gradually increased so that the jet transitions from the detached state to the attached state. For each prescribed value of δ, the instantaneous wall pressure distribution over the nozzle wall is recorded and stored as one snapshot of the pressure field. In this way, pressure fields of different jet states are all stored into a matrix. A total of N instantaneous pressure fields are acquired, each containing M pressure values for the nozzle surface. These data are assembled into a full pressure snapshot matrix:

(6) $$P_{ij}^{full}\in R^{N\times M}$$

Here, $P_{ij}^{full}$ corresponds to the wall pressure value measured at the *j*-th sensor location in the *i*-th snapshot. The superscript ‘full’ is used as a label to indicate that the temporal coefficients are obtained from the full-field pressure snapshots. Since POD is applied to the variant component of the pressure field, the mean value is removed prior to the decomposition. The mean pressure vector ${\bar{p}}_{j}\in R^{1\times M}$ is defined by averaging the pressure at each measurement location over all N snapshots:

(7) $${\bar{p}}_{j}=\frac{1}{N}\sum_{i=1}^{N}P_{ij}^{full},i=1,2,\ldots ,N$$

The centered snapshot matrix $P^{\prime }\in R^{N\times M}$ is then obtained by subtracting this mean vector from each column of $P^{full}$:

(8) $$P_{ij}^{\prime }=P_{ij}^{full}-{\bar{p}}_{j}$$

The centered snapshot matrix $P^{\prime }\in R^{N\times M}\;$ is then decomposed using singular value decomposition (SVD) to extract the POD modes:

(9) $$P^{\prime }=U\;\Sigma \;V^{T}$$

where $U\in R^{N\times r}$ and $V\in R^{M\times r}$ are orthonormal matrices, $r=rank(P^{\prime })$; $\Sigma =diag(\sigma_{1},\ldots ,\sigma_{r})\in R^{r\times r}$ contains the singular values.

The spatial POD modes are given by the columns of

(10) $$\Phi \equiv V=[\phi_{1},\phi_{2},\ldots ,\phi_{r}]\in R^{M\times r}$$

And the corresponding temporal coefficients are

(11) $$A^{full}\equiv U\;\Sigma \in R^{N\times r}$$

Thus, the centered pressure snapshots can be written as

(12) $$P^{\prime }=A^{full}\;\Phi^{T}$$

The modal energy associated with the k-th mode is proportional to $\sigma_{k}^{2}$, so the modes are naturally ordered by decreasing energy. In practice, only the first m dominant modes (m<M) are retained to construct a reduced modal basis:

(13) $$\Phi_{m}=[\phi_{1},\ldots ,\phi_{m}]\in R^{M\times m},A_{m}^{full}\in R^{N\times m},$$

This leads to the following low-rank approximation:

(14) $$P^{\prime }\approx A_{m}^{full}\;\Phi_{m}^{T}$$

For a given time instant (snapshot index) i, let $P_{i}^{full}\in R^{M}$ denote the instantaneous wall pressure vector (stacked over all M wall locations), and let $\bar{p}\in R^{M}$ be the mean pressure vector. In the truncated POD space, $P_{i}^{full}$ is modeled as

(15) $$P_{i}^{full}\approx \bar{p}+a_{i}^{full}\;\Phi_{m}^{T}$$

where $a_{i}^{full}\in R^{m}$ is the vector of modal coefficients for the i-th snapshot.

#### 2.2.2. Full-Field Pressure Reconstruction Based on Compressed Sensing

Using an excessively large number of sensors is impractical for real applications. For engineering practice, only finite pressures at K sensor locations are measured (K< M). Consequently, compressed sensing (CS) is used here to reconstruct the instantaneous wall pressure field from sparse measurements in the reduced POD space.

Let $S\in \{0,1{\}}^{K\times M}$ be the sampling matrix that extracts the entries of $P_{i}^{full}$ at the sensor locations, and let $P_{i}^{k}\in R^{K}$ denote the corresponding sparse measurement vector. The measurement model for the i-th snapshot is

(16) $$P_{i}^{k}=S\;(\bar{p}+\Phi_{m}\;a_{i}^{k})$$

This defines the following:

(17) $$y^{i}\equiv P_{i}^{k}-S\;P_{i}^{full}$$

The inverse problem for the i-th snapshot reduces to

(18) $$y^{i}=\Theta a_{i}^{k},\Theta \equiv S\;\Phi_{m},$$

The modal coefficients $a_{i}^{k}$ are obtained by solving an $l_{1}$-regularized sparse optimization problem:

(19) $$\underset{a_{i}^{k}}{min}\;\|a_{i}^{k}{\|}_{1}$$

Once the optimal coefficients $a_{i}^{k}$ are obtained, the full-field wall pressure of the i-th snapshot is reconstructed as follows:

(20) $${\hat{P}}_{i}^{full}=\bar{p}+a_{i}^{k}\;\Phi_{m}^{T}$$

By repeating this procedure for all snapshots i=1,2,…,N, the entire time series of wall pressure fields is reconstructed in a snapshot-by-snapshot manner.

#### 2.2.3. Sensor Layout Optimization Based on a Genetic Algorithm

For sensor layout optimization, this study employs a genetic algorithm (GA) to improve the accuracy of pressure field reconstruction under a prescribed number of sensors. The genetic algorithm is a random search and optimization algorithm inspired by the principles of natural selection and genetic variation in biological evolution. Its core idea is to simulate the survival competition and reproduction process of biological populations in a natural environment: candidate solutions are encoded as chromosomes, which undergo selection, crossover, mutation, and other genetic operations; through iterative evolution over generations, individuals with higher fitness values are gradually retained, enabling the algorithm to converge towards the optimal solution of the problem.

**Figure 6:**
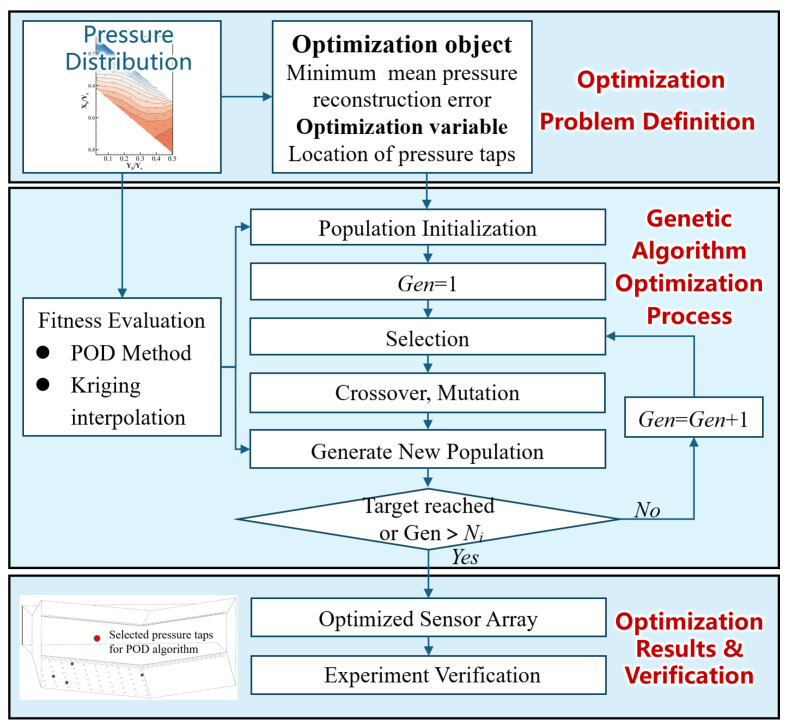
Schematic diagram of sparse pressure tap selection method based on genetic algorithm.

Figure 6 shows schematic diagram of sparse pressure tap selection method based on genetic algorithm. First, the optimization problem is defined by prescribing the number of sensors and treating the tap locations as the design variables. An initial population is then generated by randomly sampling feasible chromosomes from the candidate set. Each chromosome is evaluated by a reconstruction fitness function. With the fitness values computed, the GA transfers advantageous tap configurations to the next generation. The final output is the optimized sensor array, guaranteeing that the mean pressure reconstruction error is the minimum.

The parameter configuration is fundamentally a trade-off between convergence speed, global search capability, result stability, and computational cost. An adopted binary encoding is used for sensor layout optimization, where each individual in the population is represented by a binary string with the constraint $\sum x_{j}=k$. For crossover, the Count Preserving Crossover (CPC) was employed: coincident selected positions between parent individuals are inherited directly, and additional positions required to meet the k-sensor constraint are randomly sampled from valid candidate positions, ensuring all offspring maintain the fixed sensor count. Mutation was implemented via random flip: one selected sensor position is deselected, and a new valid position is randomly selected to maintain the k-sensor constraint, preventing premature convergence. The specific parameters were determined as follows: population size = 100, number of generations = 100, crossover probability = 0.85, mutation probability = 0.1, elitism rate = 0.1, and tournament selection with T = 3. For one sensor condition, all configurations were searched without using the genetic algorithm.

## 3. Results and Discussion

### 3.1. Characteristics of the Pressure Field

**Figure 7:**
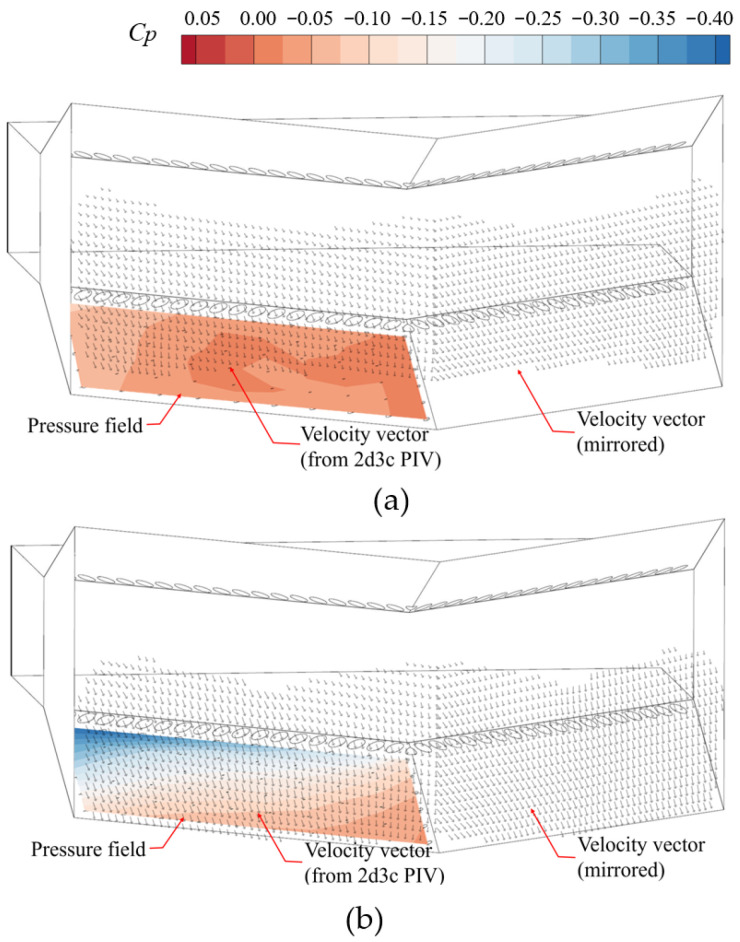
Pressure field of Coanda wall and spatial velocity distribution of main flow; (**a**) *δ* = 0.0 (neutral); (**b**) *δ* = 1.0 (attached). (The left velocity vector is the time-averaged value of PIV experimental result, and the right part is its mirror; the pressure distribution is the instant value).

In this section, the correlation between the displacement of the secondary flow channel valve and the wall pressure distribution is demonstrated through experimental results. To better visualize the three-dimensional flow characteristics, 2D3C particle image velocimetry (PIV) is employed to show the velocity distribution at the outlet of the wedge passive fluidic thrust vectoring nozzle (spatial contour). These measurements are then compared with the wall pressure under corresponding operating conditions. The laser sheet of 2D3C PIV was on the left part of the nozzle outlet plane. Two cameras were used to capture particle movement from different views, which were used to reconstruct the 3-component velocity field. A total of 20 images were acquired per condition and processed via statistical averaging to obtain flow-field data. Figure 7 shows the velocity vectors whose magnitudes are larger than 60% of the main flow speed, and the vector distributions in space can indicate the deflection state of the jet.

When the jet enters a neutral or detached state, the overall pressure distribution is smooth (Figure 7a). A low-pressure region exists near the intersection between the leading edge of the Coanda surface and the sidewall, where the pressure coefficient is approximately −0.1. An adverse pressure gradient is established from the side corner toward the intersection between the trailing edge of the Coanda surface and the central symmetry plane.

Once the jet deflects toward the Coanda surface, the jet deflection appears near the sidewall first, creating a localized low-pressure region between the leading edge of the Coanda surface and the sidewall. In contrast, the deflection angle near the central symmetry plane is smaller, with the suction intensity gradually weakening from the sidewall toward the centerline. As the opening difference in the secondary flow valves further increases, the jet deflection increases, causing this low-pressure region to expand. When the jet near the sidewall achieves a fully deflected state (Figure 7b), the minimum pressure coefficient in that vicinity is approximately −0.4.

### 3.2. POD Model Training Results

**Figure 8:**
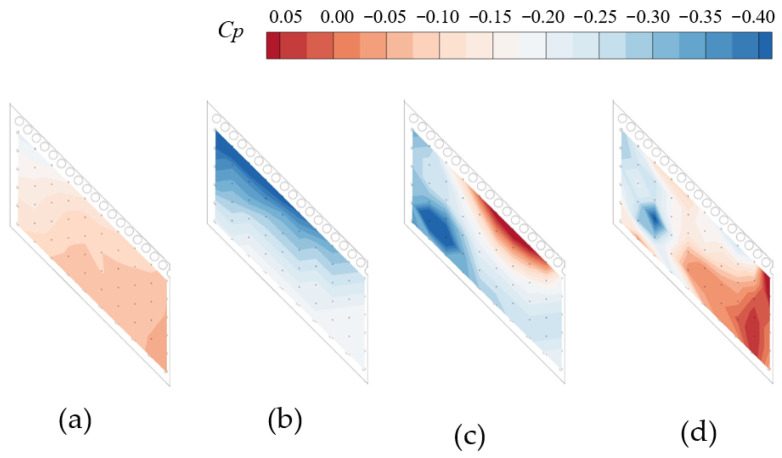
POD modal decomposition results of pressure measured on the down side of the Coanda wall. (**a**) Mean value; (**b**) Mode 1; (**c**) Mode 2; (**d**) Mode 3.

Based on the training snapshots, singular value decomposition (SVD) is applied to extract the POD modes. Figure 8a shows the mean wall pressure distribution, which is approximately the mean pressure on the Coanda surface when the jet is in the detached state. The three POD modes occupy 97.5%, 2.0%, and 0.3% of the total energy. Figure 8b presents the first mode, which primarily characterizes the strong suction region near the leading edge of the Coanda surface after jet attachment; the suction intensity gradually decreases from the nozzle sidewall toward the center symmetry plane. Figure 8c shows the second mode, which complements the first mode by highlighting the intense suction in the vicinity of the sidewall together with the recovery of a pressure plateau near the symmetry plane. Figure 8d corresponds to the third mode, whose energy contribution is relatively small and can be regarded as a minor correction to the first two modes. According to the 99.5% cumulative energy criterion, the first (k = 2) dominant modes are retained, which are sufficient to capture the essential features of the original pressure field.

### 3.3. Sparse Reconstruction Results

The genetic algorithm is used to optimize the layout of the wall pressure sensors for each prescribed sensor number, with the objective of minimizing the mean reconstruction error. To evaluate reconstruction performance over the entire Coanda wall under different jet deflection states, two normalized error indices, f(x) and g(x), are defined for a given sensor layout x**.**

Let i=1,…,N denote the snapshot index; each snapshot corresponds to a specific jet deflection state, and j=1,…,M denotes the index of wall pressure measurement points. The reference and reconstructed wall pressures at the j-th location of the i-th snapshot are denoted by $P_{ij}^{\mathrm{full}}$ and ${\hat{P}}_{ij}^{\mathrm{full}}$. The absolute reconstruction error at the *j*-th location of the *i*-th snapshot is defined as

(21) $$\varepsilon_{ij}=|P_{ij}^{\mathrm{full}}-{\hat{P}}_{ij}^{\mathrm{full}}|$$

For a given sensor layout x, the normalized mean error m(x,i) and normalized maximum error h(x,i) over all measurement points for the i-th snapshot are defined as

(22) $$m\left(x,i\right)=\frac{\frac{1}{M}\sum_{j=1}^{M}\;\left|\varepsilon_{ij}\right|}{G},h(x,i)=\frac{\underset{1\leq j\leq M}{max}\left(\varepsilon_{ij}\right)}{G}$$

where $G=\underset{1\leq i\leq N,1\leq j\leq M}{max}\left(\left|P_{ij}^{full}\right|\right)$ represents the global maximum absolute pressure from the reference dataset. Finally, the two normalized error indices for the sensor layout x are defined as the maximum values of m(x,i) and h(x,i) over all snapshots, respectively:

(23) $$f\left(x\right)=\underset{1\leq i\leq N}{max}\left(m\left(x,i\right)\right),g\left(x\right)=\underset{1\leq i\leq N}{max}\left(h(x,i)\right)$$

The number of sensors is prescribed in the range of 1–15. The optimization objective of the genetic algorithm can be expressed as follows:

(24) $$\underset{x}{min\;}f(x)$$

**Figure 9:**
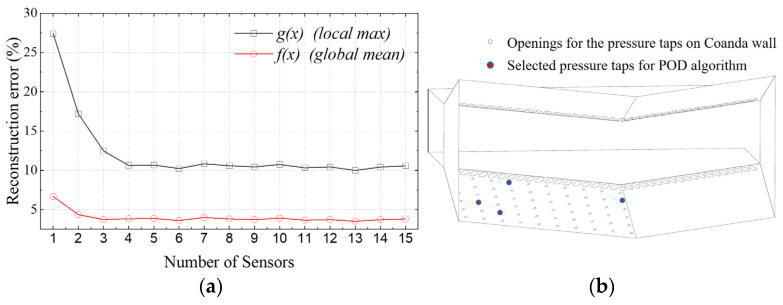
Optimized sensor layout and pressure reconstruction error variation with pressure tap number. (**a**) Variation in the f(x) and g(x) with the number of Coanda wall pressure taps; (**b**) the optimized sensor layout for POD algorithm of four pressure taps.

Figure 9a shows the influence of the number of pressure taps on the f(x) and g(x) with the number of sensors. For each prescribed number of pressure taps, the genetic algorithm provides the optimal layout and the corresponding reconstruction error. Both the error f(x) and g(x) decrease rapidly when the number of sensors increases from 1 to about 4; According to Figure 9a, the error g(x) drops from 27.4% to 10.6%, while the error f(x) is reduced to below 4%. As the number of sensors further increases, the curves gradually approach a plateau, indicating that additional sensors bring only marginal improvements in reconstruction accuracy. This trend suggests that a pressure tap count of four offers a good compromise between reconstruction performance and system complexity. And the optimized sensor layout is shown in Figure 9b.

**Figure 10:**
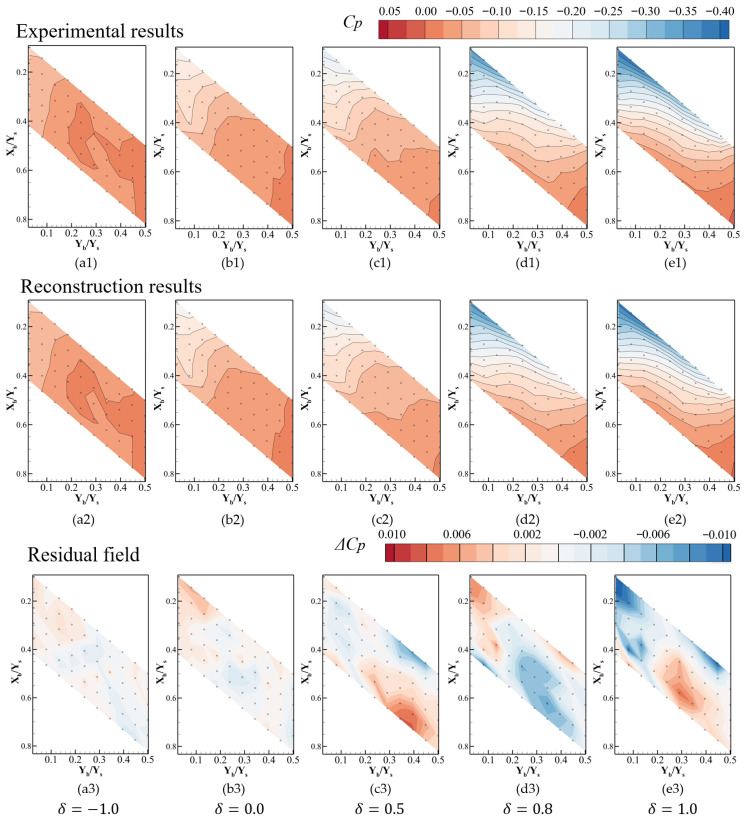
Comparison of pressure field reconstruction at typical jet deflection states, (**a1**–**a3**) *δ* = −1.0 (detached), (**b1**–**b3**) *δ* = 0.0 (neutral), (**c1**–**c3**) *δ* = 0.5, (**d1**–**d3**) *δ* = 0.8, (**e1**–**e3**) *δ* = 1.0 (attached).

Figure 10 compares the experimentally measured Coanda wall pressure distributions with the POD-based reconstructions for different jet deflection states. In each block, subplots (1), (2), and (3) correspond to the experimental contours of $C_{p}$, the reconstructed ${\hat{C}}_{p}$ obtained from the POD-based method, and the reconstruction error $\left|\Delta C_{p}\right|=C_{p}-{\hat{C}}_{p}$, respectively. The rows (a)–(e) represent increasing values of the control parameter δ.

For δ=−1 and δ=0, the jet deflection state is detached or neutral, and the pressure field is dominated by a relatively mild streamwise gradient, with a slightly lower pressure near the upstream corner and higher pressure toward the downstream mirror plane. The POD reconstruction reproduces these smooth distributions. The corresponding error maps (a3, b3) show that the $\left|\Delta C_{p}\right|$ is less than 0.02. As δ increases to 0.5 and 0.8, the jet progressively attaches to the Coanda wall, and a pronounced low-pressure region develops along the upper, upstream portion of the inclined surface. This manifests as a blue band of decreased $C_{p}$ in the experimental fields (c1, d1), accompanied by steeper pressure gradients across the upstream region. The POD-based reconstructions (c2, d2) capture both the position and extent of this low-pressure band, as well as the downstream pressure recovery toward a higher $C_{p}$. The main features match well, indicating that the dominant flow structures associated with jet attachment are correctly reconstructed. At δ=1, corresponding to the maximum jet deflection, the low-pressure zone extends over a wider upstream area, and the overall pressure level on the Coanda wall is significantly reduced compared with the neutral and detached cases. The residual field (e3) remains dominated by small-amplitude, localized patches, confirming that the proposed POD-based method maintains a good accuracy across the entire range of jet deflection conditions.

**Figure 11:**
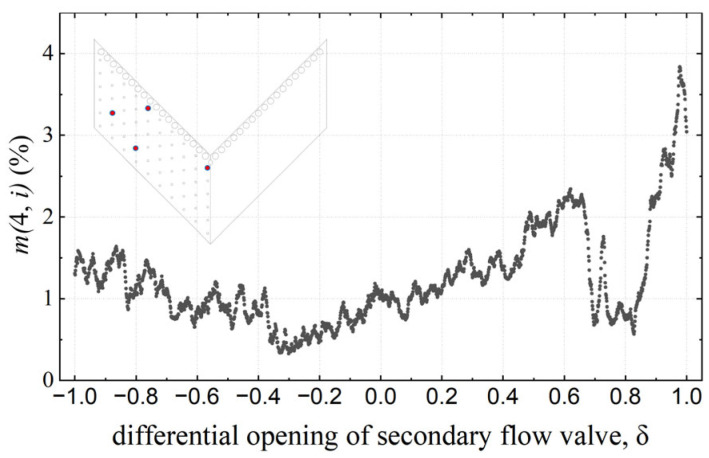
Variation in the mean error m(4,i) of *i*-th snapshot with the differential opening of secondary flow valves, *δ*. Red dots are selected pressure taps.

To quantify the normalized mean error of pressure reconstruction with different jet deflection states, the m(4,i) of different jet deflection states is calculated. Figure 11 presents the mean error m(4,i) under different jet deflection states. In the detached and neutral states (*δ* ≤ 0), the maximum error is within 1.6%; meanwhile, the wall pressure distribution changes slightly and is close to the average pressure value. When the jet begins to attach to the wall (in the range of δ = 0–0.6), the maximum pressure error rises steadily, indicating that the pressure gradually deviates from the original working condition and increases with the deviation of the pressure distribution from the average value. In the range of δ = 0.6–1.0, the error fluctuates sharply and decreases first, and then rises. From the perspective of pressure distribution (as shown in Figure 10), when δ = 0.8, a low-pressure region appears at the leading edge, which is close to the pressure distribution of Mode 1. At this point, the effect of utilizing Mode 1 is significant. According to the results in Figure 10(d3), the pressure error is positive near the side plates but negative near the symmetry plane. When δ = 1.0, as shown in Figure 10(e3), the pressure error is negative near the side plates but positive near the symmetry plane. Compared with δ = 0.8, the sign of the error switches, meaning the overfitting occurs. The maximum m(4,i) is within 4%.

### 3.4. Comparation with Kriging Method

**Figure 12:**
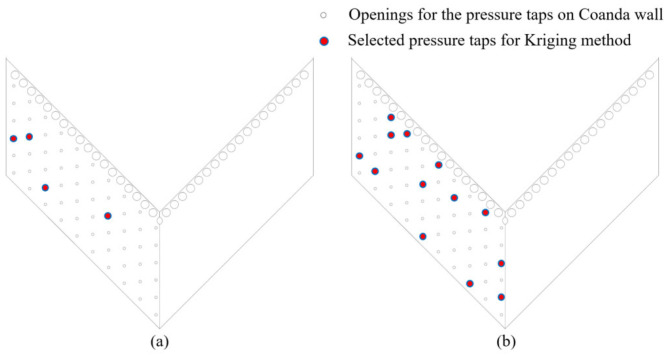
The optimized sensor layout for 4 and 13 pressure taps for Kriging algorithm: (**a**) 4 pressure taps; (**b**) 13 pressure taps.

**Figure 13:**
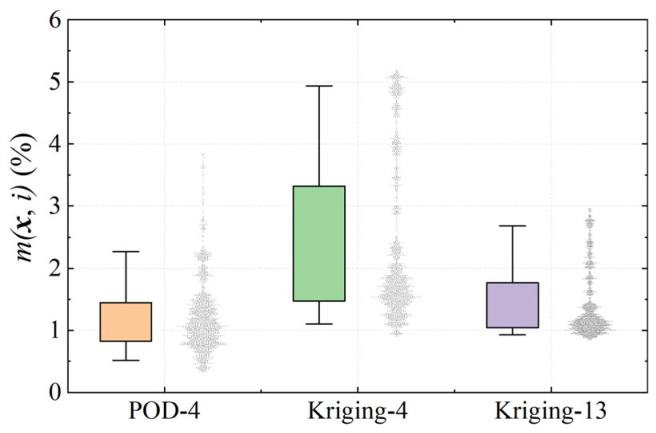
Reconstruction error for POD and Kriging methods using 4 and 13 sensors.

To further evaluate the reconstruction capability of the proposed method, the POD-based approach is compared with conventional Kriging interpolation. Figure 12 illustrates the optimized sensor layouts of 4 and 13 sensors for Kriging interpolation, where the sensor layout optimization is implemented using the genetic algorithm. Figure 13 presents a comparison of reconstruction errors between the POD and Kriging methods with the deployment of 4 and 13 sensors, respectively. In Figure 13, each box represents the 25–75% of m(x,i), while the whiskers indicate the 5–95 percent, corresponding to an interval of approximately 4σ of the error distribution. The reconstruction errors of single snapshots were drawn as dots on the right of the box.

With four sensors, the POD reconstruction errors are tightly concentrated around 1%, and the associated dispersion is small. In contrast, the four-sensor Kriging scheme exhibits larger error clusters and a much broader spread, indicating a poorer robustness. When the number of sensors for Kriging is increased to 13, the interquartile range becomes comparable to that of the 4-sensor POD case.

**Figure 14:**
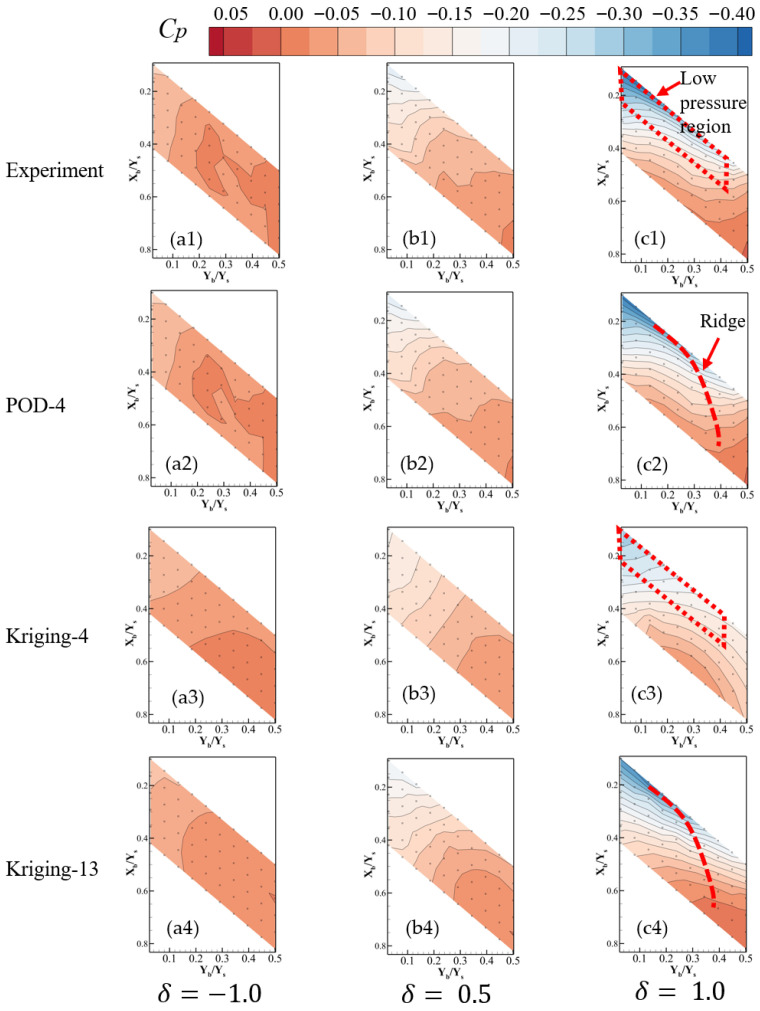
Comparison of reconstruction pressure field for POD and Kriging methods using 4 and 13 sensors. (**a1**–**a4**) *δ* = −1.0 (detached), (**b1**–**b4**) *δ* = 0.5, (**c1**–**c4**) *δ* = 1.0 (attached).

To further elucidate the mechanism underlying the reconstruction errors, pressure contour maps obtained from the three reconstruction algorithms are presented in Figure 14. Figure 14(a1–c1,a2–c2,a3–c3,a4–c4) correspond to the experimental results, POD-4, Kriging-4, and Kriging-13 results. The POD-based reconstruction results have been introduced in Section 3.3. Although the sensor locations for the Kriging-4 method are optimized by the genetic algorithm, the reconstructed fields still fail to capture the essential pressure field features. In particular, at δ = 1.0, as shown in Figure 14, for the experimental data (c1), the pressure in the leading-edge low-pressure region is noticeably lower than the surrounding values. For the POD-4 case (c2), the low-pressure structure is recovered, while, for the Kriging-4 case (c3), this region is absent. Moreover, a ridge of the pressure suction line is also absent for the Kriging-4 case. For the Kriging-13 case, the pressure field structure is recovered, which means the Kriging method needs more sensors for high-fidelity pressure field feature reconstruction.

## 4. Conclusions

In this work, a sparse reconstruction of the pressure field method based on the POD algorithm has been developed for the wedge passive fluidic thrust vectoring nozzle. The methodology is validated on a low-speed jet platform. The main conclusions are summarized as follows:

1. Pressure field snapshots measured on the Coanda surface of various jet deflection statuses are decomposed using proper orthogonal decomposition (POD). The first two dominant modes are found to capture approximately 99.5% of the cumulative modal energy, which is sufficient to represent the key three-dimensional features of the pressure field.
2. By combining the POD basis with an $l_{1}$-regularized compressed sensing formulation, the full Coanda wall pressure distribution is reconstructed from a limited set of pressure sensors. A genetic algorithm is employed to optimize the locations of pressure taps for a prescribed number of sensors. The reconstruction accuracy improves rapidly as the sensor count increases from one to four for the POD algorithm, after which the error curves gradually approach a plateau.
3. The reconstructed fields closely matched the experimental measurements over the entire range of jet deflection conditions with only four sensors. The local reconstruction error remained within $|\Delta C_{p}|<0.02\;$ across the Coanda surface.
4. In contrast, Kriging interpolation required increasing the sensor count to 13 to achieve comparable results to the POD-4 case, yet still exhibited larger errors and less fidelity in the structure of the pressure field.

Overall, the proposed sparse-sensor POD reconstruction algorithm provides an efficient and physically interpretable strategy to estimate the pressure distribution using sparse pressure measurements. The advantage of this method lies in its ability to achieve a high-precision reconstruction of pressure distributions based solely on sparse pressure point information for pressure fields with prominent three-dimensional pressure distribution characteristics. Future work will perform the following:

1. Evaluate the application effect of the proposed pressure field reconstruction algorithm under different inflow velocities;
2. Predict the aerodynamic forces acting on the fluidic thrust vectoring nozzle using the reconstructed pressure field;
3. Estimate the dynamic pressure field;
4. Integrate algorithms such as Kalman filtering to enhance the robustness of the proposed algorithm under dynamic conditions and noise interference.

## Figures and Tables

**Table 1:** Dimensionless position coordinates of pressure tap on nozzle wall.

Axis	$X_{b}/L_{c}$	${2Y}_{b}/Y_{s}$
Value	0.10, 0.26, 0.42, 0.58, 0.74, 0.90	0.05, 0.15, 0.25, 0.35, 0.45 0.55, 0.65, 0.75, 0.85, 0.95

**Table 2:** Key geometric parameters of the nozzle.

Parameter	Symbol	Accuracy
Coanda Wall Deflection Angle	θ	$15^{\circ }$
Nozzle Exit Width	$Y_{n}$	150 mm
Nozzle Inlet Height	$H_{1}$	30 mm
Nozzle Outlet Height	$H_{2}$	67.08 mm
Coanda Wall Chord Length	$L_{c}$	60 mm

## Data Availability

The original contributions presented in this study are included in the article. Further inquiries can be directed to the corresponding author.

## Nomenclature

δ	The differential opening of secondary flow valves
$X_{b}$	X coordinates of pressure tap
$L_{c}$	Coanda wall chord length
$Y_{b}$	Y coordinates of pressure tap
$Y_{s}$	Twice the length of the nozzle trailing edge
$P_{i}$	Pressure measured by the pressure sensor
$P_{s}$	Surface static pressure
$P_{a}$	Atmospheric pressure
$C_{p}$	Pressure coefficients
N	Number of snapshots
M	Number of wall locations for pressure measurement
$P_{ij}^{full}$	Wall pressure value measured at the *j*-th sensor location in the *i*-th snapshot
${\bar{p}}_{j}$	Mean pressure vector
$P^{\prime }$	Centered snapshot matrix
U , V	Orthonormal matrix obtained from singular value decomposition
r	$\mathrm{Rank}\;\mathrm{of}\;\mathrm{the}\;\mathrm{centered}\;\mathrm{snapshot}\;\mathrm{matrix}\;P^{\prime }$
Σ	Diagonal matrix containing singular values
$\sigma_{r}$	Element of matrix Σ
Φ	Matrix of spatial POD modes
$\phi_{r}$	Individual POD modes
$A^{full}$	Matrix of temporal coefficients corresponding to full POD modes
m	Number of retained dominant POD modes
$\Phi_{m}$	Reduced modal basis composed of the first m dominant POD modes
$A_{m}^{full}$	Matrix of temporal coefficients corresponding to the reduced modal basis
$P_{i}^{full}$	Instantaneous wall pressure vector
$a_{i}^{full}$	Vector of modal coefficients for the *i*-th snapshot
K	Number of finite sensor locations
S	Sampling matrix
$P_{i}^{k}$	Sparse measurement vector corresponding to the *i*-th snapshot
$y^{i}$	$\mathrm{Defined}\;\mathrm{vector},\;\mathrm{equal}\;\mathrm{to}\;P_{i}^{k}-S\;P_{i}^{full}$
Θ	$\mathrm{Matrix}\;\mathrm{defined}\;\mathrm{as}\;S\;\Phi_{m}$
$a_{i}^{k}$	Modal coefficients in the compressed sensing framework
${\hat{P}}_{i}^{full}$	Reconstructed full-field wall pressure vector
x	Sensor layout
f(x)	Normalized error index, represents local max error
g(x)	Normalized error index, represents global mean error
m(x,i)	Normalized mean error over all measurement points (for the i-th snapshot under given sensor layout x)
h(x,i)	Normalized maximum error over all measurement points (for the i-th snapshot under given sensor layout x)
G	Global maximum absolute pressure from the reference dataset
${\hat{C}}_{p}$	Reconstructed pressure coefficients
$\Delta C_{p}$	Reconstruction pressure coefficient error
